# The spectrum and differential diagnosis of acquired ocular motor nerve palsies: a clinical study of 502 patients

**DOI:** 10.1007/s00415-021-10761-w

**Published:** 2021-09-19

**Authors:** Rebecca Hörner, Jan Kassubek, Jens Dreyhaupt, Albert C. Ludolph

**Affiliations:** 1 Department of Neurology, University of Ulm, Oberer Eselsberg 45, 89081 Ulm, Germany; 2 Institute of Epidemiology and Medical Biometry, University of Ulm, Ulm, Germany

**Keywords:** Cranial nerve palsy, Ocular motor nerve, Trochlear nerve, Abducens nerve, Magnetic resonance imaging, Cerebrospinal fluid

## Abstract

**Background:**

Ocular motor nerve palsies (OMNP) frequently cause patients to present in an emergency room. In the following study, we report the differential diagnosis of OMNP by use of magnetic resonance imaging (MRI) and CSF examination as a standard.

**Method:**

We performed a data analysis of *N* = 502 patients who presented with oculomotor, trochlear, and/or abducens nerve palsy in the emergency room of the Department of Neurology, University of Ulm, between January 2006 and December 2019. We report clinical and MRI scan findings in all patients; furthermore, the CSF of 398 patients has been analysed.

**Results:**

Abducens nerve palsies were most common (45%), followed by palsies of the oculomotor (31%) (CNP III) and trochlear nerve (15%). Multiple OMNPs were seen in 9% of our cohort. The most common causes included inflammations (32.7%), space-occupying lesions, such as aneurysms or neoplasms (17.3%), diabetes mellitus (13.3%), and brainstem infarctions (11%). Still 23.4% of the patients could not be assigned to any specific cause after differential diagnostic procedures and were described as idiopathic. One of three patients with an inflammation and 39% of the patients with space-occupying lesions showed additional cranial nerve deficits.

**Conclusion:**

Inflammation and space-occupying processes were the most frequent causes of OMNP, although brainstem infarctions also play a significant role, in particular in CNP III. The presence of additional CNPs increases the probability of an inflammatory or space-occupying cause.

## Introduction

When ocular motor nerve palsies (OMNP) cause patients to present in a neurological clinic, a broad differential diagnostic procedure is necessary to distinguish between the various heterogeneous etiologies. They include a wide range of causes such as inflammations, space-occupying lesions such as neoplasms and aneurysms, ischemia or trauma [1–6]. According to the literature, the abducens nerve (CNP VI) is the second most affected cranial nerve (CNP) after the facial nerve with an annual incidence of about 11.3/100000 [7]. In most previous studies the mere presence of risk factors—such as diabetes, hypertension, old age or obesity—was considered to be sufficient to assign the palsies to a cause, in particular of vascular origin. Large retrospective studies with 4278 patients date back to the 1990s [3] and could not take advantage of currently broadly available diagnostic tools. Diagnostic procedures such as MRI-based neuroimaging and, if possible, CSF analysis offer a more straightforward opportunity to define the etiology of OMNP [8]. In the literature, there is large variation in the reported etiologies of OMNP, e.g., the frequency of neoplasms causing oculomotor nerve palsies varies between 1 and 13% in previous reports [1, 4, 6, 9]. In addition, according to the available literature, between 16 and 31% of all OMNP could not be assigned to a specific etiology and were, therefore, defined as idiopathic [1, 4, 6, 10]. In this study of 502 OMNP patients, we analyzed data in the setting of a Neurological University Clinic where state of the art MRI techniques and, if necessary, detailed CSF studies were prospectively used for differential diagnosis.

## Methods

We analyzed 502 patients with acquired ocular motor nerve palsies (OMNP) admitted to the Department of Neurology, University of Ulm between January 2006 and December 2019 according to a standardized prospective protocol. Patients were included in the study (inclusion criteria) if they presented with an acute clinical picture of an OMNP and if a routine procedure of a MRI exam and lumbar puncture with an analysis of the cerebrospinal fluid was conducted. Congenital or long-standing OMNPs were not included in the study. Each patient received a complete clinic-neurological examination by a board-certified neurologist. The diagnosis of OMNP was based on a clinical motility test of the individual muscles. In rare cases an orthoptist was involved if the palsy was difficult to classify, this was particularly true for suspected trochlear palsies. Furthermore, a complete medical history was provided, which included comorbidities (such as diabetes, hypertension, and obesity) and other symptoms such as headache, ocular pain, and previous infections. Specific focus in the history was put on the timing of the initial symptoms of the palsy (insidiously, suddenly). In all 502 patients, magnetic resonance imaging (MRI) was done, and 398 patients underwent lumbar puncture for CSF examination. In the remaining 104 patients only a cMRI scan was performed, since neuroimaging already revealed a definitive cause of the palsy in 76 patients such as an ischemic lesion or a neoplasm. In the remaining 28 patients, the MRI did not unequivocally reveal a specific cause of the palsy, but the patient did not consent in a lumbar puncture. According to the definition of an idiopathic palsy, these patients cannot be assigned to this category, given that a CSF examination would have been necessary to rule out other causes. For this reason, these 28 patients were only included in the evaluation of the epidemiologic and demographic results and were not assigned to one of the etiological categories. This study was performed in compliance with ethics committee standards at the University of Ulm.

All 502 patients received imaging of the brain by use of a 1.5 T MRI scanner (Magnetom TIM Symphony, Siemens, Erlangen, Germany) equipped with a 12-channel head coil. The standard MRI protocol consisted of the following sequences: 2D transaxial T1-weighted spin echo sequence, transaxial 2D dual-echo proton density-weighted/T2-weighted turbo spin echo, transaxial diffusion-weighted imaging (DWI) with apparent diffusion coefficient (ADC) mapping, transaxial T2*-weighted gradient echo sequence (fast low angle shot), and a coronar fluid-attenuated inversion recovery (FLAIR) sequence, together with a time-of-flight magnetic resonance angiography (TOF-MRA) of the brain-supplying arteries. To increase the diagnostic information in specific patients, additional T1-weighted sequences with the contrast agent gadoteridol in a dose of 0.1–0.3 mmol/kg body weight were acquired. In addition, additional sequences were optionally acquired, such as fat-saturated orbita sequences, and specific brainstem imaging such as constructive interference steady state (CISS) sequences.

398 of the 502 patients additionally underwent a lumbar puncture to analyse CSF parameters including white cell count (per µl), total protein (mg/l), lactate (mmol/l), CSF/serum albumin ratio (× 10^–3^), oligoclonal IgG bands in CSF and antibody indices to varicella-zoster-virus (VZV), herpes-simplex-virus (HSV 1 and 2) or Borrelia Afzelii in combination with measurement of CXCL13 (cut off > 300 pg/ml). Specific antibody indices (AI = (CSF/serum ratio IgG VZV)/(CSF/serum ratio total IgG)) were classified positive when AI ≥ 1.5. Furthermore, the CSF opening pressure was measured to rule out intracranial hypertension.

After etiological work-up of the OMNPs, our cohort was divided into seven categories:

- Patients with space-occupying lesions: processes in proximity to the cranial nerve (CN) or its nucleus. If the CSF showed cells indicative of neoplastic meningeosis, these patients were also included in this group.
- Patients with autoimmune/inflammatory diseases: These included autoimmune diseases (e.g., Multiple sclerosis, Miller-Fisher Syndrome, Myasthenia gravis) and other inflammatory processes (e.g., Tolosa–Hunt syndrome).
- Patients with viral/bacterial infections: This category includes proven viral and bacterial infections, such as meningoencephalitis or infections of neighbouring tissues such as sinuses or inflammations of the orbita with secondary affection of the respective nerve.
- Patients with brainstem infarctions: Ischemic lesions of the mesencephalon or pons as shown by MRI.
- Patients with diabetes mellitus: Presence of diabetes mellitus Type 1 or 2 and no other explanation for the oculomotor deficit.
- Patients with other etiologies: Rare causes or causes which could not be assigned to another category.
- Idiopathic palsy: no underlying cause could be found.

**Fig. 1:**
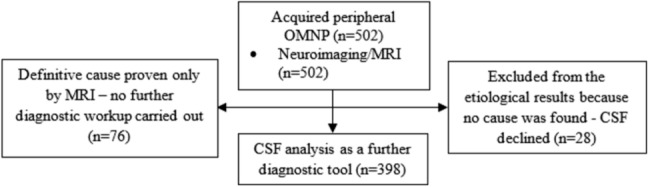
Study flow diagram

Figure 1 shows the study flow diagram. Initially, 517 patients with acquired OMNP were admitted to the emergency room with the suspected diagnosis of an oculomotor palsy. We excluded 15 patients with central palsies and included the remaining 502 patients into our study.

Statistical analyses were performed using SAS, version 9.4 (SAS Institute, Chicago, IL, USA). Continuous variables were described by mean and standard deviation. Absolute frequencies and percentages were used to describe categorical variables. We carried out the chi square test for comparisons in categorical variables. A test result was considered significant if the two-sided *p* value was < 0.05. Due to the explorative nature of our study, all results from statistical tests must be interpreted as hypothesis generating. An adjustment for multiple testing was not done.

## Results

### Demography and epidemiology

**Fig. 2:**
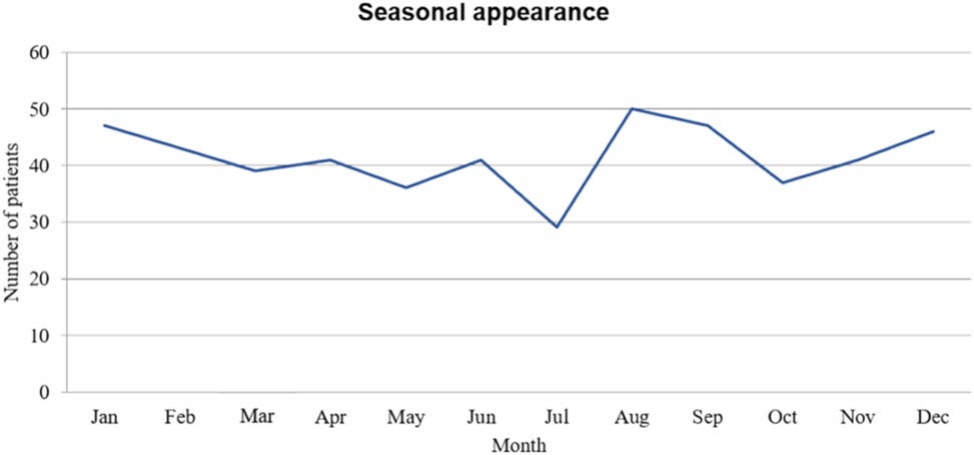
Seasonal appearance of the OMNPs in our study. A peak seemed to be present in late summer

The total cohort of 502 patients was included in the description of epidemiologic and demographic data. 90.8% (*n* = 456) showed isolated acquired OMNP and 9.2% had multiple OMNPs (*n* = 46). The cohort comprised 273 men (54.4%) and 229 women (45.6%) with a mean age of 61.2 years (SD 17.5) and an age range of 16–92 years. The most common isolated OMNP was CNP VI (44.6%), followed by oculomotor nerve palsy with 31.3% (CNP III) and trochlear nerve palsies (CNP IV) with 14.9% (refer to Table 1). We saw a seasonal peak in August and September (Fig. 2). The medical history of 84 of 502 patients (16.7%) revealed previous OMNP earlier in life. Comorbidities were not uncommon in patients with OMNP, as shown in Table 1, specifically, 23.9% had diabetes, 57.4% had arterial hypertension, and 20.9% had severe obesity (BMI ≥ 30).

**Table 1:** Demographic description of our cohort and the association of palsies with hypertension, diabetes and obesity

	CNP III	CNP IV	CNP VI	Multiple	Total
Patients, *n* (%)	157 (31.3)	75 (14.9)	224 (44.6)	46 (9.1)	502 (100)
Age, yr	64.9 ± 16.3	59.9 ± 15.1	59.3 ± 18.6	59.8 ± 17.9	61.2 ± 17.5
Male/female	86/71	47/28	120/104	20/26	273/229
Hypertension, *n* (%)	98 (62.4)	45 (60)	123 (54.9)	22 (47.8)	288 (57.4)
Diabetes, *n* (%)	53 (33.8)	20 (26.7)	42 (18.8)	5 (10.9)	120 (23.9)
Obesity, *n* (%)	37 (23.6)	11 (14.7)	46 (20.5)	11 (23.9)	105 (20.9)

### Etiology

The most common causes were space-occupying lesions (17.3%), autoimmune/inflammatory processes (25.7%), viral/bacterial infections (6.3%), brainstem infarctions (11%), and diabetic (13.3%) or idiopathic (23.4%) palsies. The frequency of brainstem infarctions was somewhat higher in CNP III (19.7%) than in the other OMNPs. The frequency of diabetogenic and idiopathic palsies was higher in CNP IV compared to the CNP III and CNP VI, but only the difference between the idiopathic CNP IV and CNP III was statistically significant (*p* = 0.0001). The main cause of multiple OMNPs was inflammation (67.4%). Table 2 shows the distribution of etiologies across the different CNPs.

**Table 2:** Proportion of etiological subgroups in CNP III, CNP IV, CNP VI, and multiple OMNP

Etiologies	CNP III ≈% (*n* = 147)	CNP IV ≈% (*n* = 71)	CNP VI ≈% (*n* = 210)	Multiple ≈% (*n* = 46)
Idiopathic	15.6% (23)	39.4% (28)	27.1% (57)	6.5% (3)
Diabetogenic	13.6% (20)	21.1% (15)	13.3% (28)	0
Space-occupying lesions thereof:	20.4% (30)	2.8% (2)	18.6% (39)	23.9% (11)
–Intraorbital	0.7% (1)	0	1.0% (2)	4.3% (2)
–Lesions close to the cavernous sinus	9.5% (14)	1.4% (1)	4.8% (10)	13.0% (6)
–Intracerebral, meningeal, intrathecal	10.2% (15)	1.4% (1)	12.9% (27)	6.5% (3)
Inflammation thereof:	27.2% (40)	25.4% (18)	30% (63)	67.4% (31)
–Autoimmune/inflammatory	22.4% (33)	19.7% (14)	23.8% (50)	54.3% (25)
–Viral, bacterial	4.8% (7)	5.6% (4)	6.2% (13)	13.0% (6)
Brainstem infarctions	19.7% (29)	9.9% (7)	7.1% (15)	2.2% (1)
Others	3.4% (5)	1.4% (1)	3.8% (8)	0

### Oculomotor nerve palsies (CNP III)

One hundred and forty-seven patients presented with CNP III; 72.8% of the palsies were incomplete (*n* = 107). A ptosis occurred in 69.3%, a mydriasis in 34.3%. Twenty-three of 147 patients (15.6%) were diagnosed with idiopathic CNP III, among them were 73.9% incomplete palsies. The information regarding the onset of the palsy is based on the report of the patients when their history was taken by a physician. Here, 13 patients (56.5%) in this group described a sudden onset of the palsy (69.6% of these patients also had a ptosis, 21.7% a mydriasis), in the remaining 44.5% the onset was not described as sudden. 20 of 147 patients (13.6%) with CNP III were classified as diabetogenic, 80% of them were incomplete. Of those palsies ascribed to diabetes, 16 patients (80%) had a sudden onset of symptoms. A ptosis occurred in 80% of all idiopathic palsies, a mydriasis in 33.3%.

Thirty patients with CNP III (20.4%) were diagnosed with space-occupying lesions. These lesions represented the second most common cause of CNP III in our cohort. The ratio between incomplete and complete palsies was 15:15. 46.7% of all space-occupying lesions were found in close neighborhood to the cavernous sinus. One tumor was found inside the orbita, the remaining lesions were found intracerebrally, close to the meninges or intrathecally. The most common space-occupying lesions were pituitary macroadenomas (*n* = 7), followed by aneurysms (*n* = 6) and meningeosis carcinomatosa (*n* = 4); other causes were meningioma (*n* = 3), cavernous-sinus-thrombosis (*n* = 2), thalamic and pontine bleedings (*n* = 2), metastases (*n* = 1), brainstem tumour (*n* = 1), intracranial hypertension (*n* = 1), and a sphenoidal mucous cyst (*n* = 1). In this category 93.3% also had a ptosis and 55.6% mydriasis. Furthermore, one palsy was also caused by a cavernous-sinus-fistula (*n* = 1). These patients with a space-occupying lesion often did not present with a palsy alone. For example, we found out that 84.6% of all patients with pituitary macroadenoma (*N* = 13 in all OMNP together) had additional symptoms such as visual field loss or headache.

Inflammations were the most common etiologies of CNP III (27.2%): 33 palsies occurred due to autoimmune/inflammatory processes and seven palsies due to viral or bacterial infections. Autoimmune/inflammatory disorders comprised Tolosa–Hunt syndrome (*n* = 13), ocular myasthenia gravis (*n* = 5; presence of acetylcholine-receptor antibodies), multiple sclerosis (*n* = 4), Miller-Fisher syndrome (*n* = 3), giant-cell arteritis (*n* = 3) and less common diseases such as Morbus Wegener (granulomatosis with polyangiitis; high titer of cANCA) and systemic lupus erythematosus. The most common defined pathogen was the varicella-zoster-virus (*n* = 4). The remaining patients had pneumococcal meningitis (with typical symptoms plus OMNP) and sinusitis with secondary orbital involvement. In this category, incomplete CNPs III dominated with 70% of these patients (ptosis occurred in 85% and mydriasis in 29.4%).

The cause of 29 of 147 CNP III palsies were brainstem infarctions as identified by MRI. Most of the palsies (86.2%) were incomplete, 27 patients (93.1%) described a sudden onset. A quarter of the patients also had a mydriasis (25.9%) and 62.1% ptosis. It is well-known that vascular risk factors such as diabetes and hypertension favor ischemic events; therefore, it is not surprising that almost half of the patients (48.3%) had diabetes and 79.3% of the patients had arterial hypertension. Patients with brainstem infarctions had the highest mean age among all etiologies.

We saw five patients with heterogenous causes, who could not be assigned to another subgroup. Two patients had a traumatic CNP III due to an orbital fracture, two patients suffered from recurring ophthalmoplegic migraine and one patient acquired his iatrogenic CNP III after a botulinum toxin injection. Traumatic OMNPs were very rare in our study, presumably because we analyzed a cohort in a neurological, not a (neuro-)surgical setting. Table 3 shows the mean age for each CNP and etiology.

**Table 3:** Mean age in patients with CNP III, CNP IV, CNP VI and multiple OMNP

Etiologies	Mean age, yr			
	CNP III (*N* = 147)	CNP IV (*N* = 71)	CNP VI (*N* = 210)	Multiple (*N* = 46)
Idiopathic	60.7	57.4	57.4	54.0
Diabetogenic	68.3	65.7	65.7	–
Space-occupying lesion	61.5	65.5	65.5	64.4
Inflammation	67	57.1	57.1	59.5
Brainstem infarction	68.4	62.0	62.0	39.0
Others	56.4	73.0	73.0	–

### Trochlear nerve palsies (CNP IV)

Isolated trochlear nerve palsies (CNP IV) were rare in our cohort (15%). 28 of 71 patients were classified as idiopathic CNP IV (39.4%)—the largest etiologic subgroup of CNP IV.

Inflammations (25.4%) were the second most common etiological subgroup of CNP IV. Of 18 patients, 14 had autoimmune/inflammatory diseases (77.8%) and four patients had viral or bacterial infections. The autoimmune/inflammatory diseases comprised myasthenia gravis (*n* = 5), multiple sclerosis (*n* = 3), Tolosa–Hunt syndrome (*n* = 2), paraneoplastic syndrome (*n* = 2; presence of metastatic tumours), rheumatoid arthritis (*n* = 1; positive rheumatoid factor and international criteria fulfilled), and an unclassified autoimmune process. The pathogenic infections were similar to CNP III, i.e., VZV-infections (*n* = 2), bacterial rhinosinusitis (*n* = 1), and another viral infection (*n* = 1).

The third most common etiological subgroup were palsies associated with diabetes mellitus (21.1%; 15 patients). Here, 11 patients (73.3%) reported that they experienced a sudden onset of the palsy. In only two patients (2.8%) the palsies were due to space-occupying lesions (basilar artery aneurysm and a sphenoid meningioma). Space-occupying lesions, thus, occurred significantly less in CNP IV compared to CNP III (*p* = 0.0006) or CNP VI (*p* = 0.012).

Brainstem infarctions were less common with CNP IV: only seven patients (9.9%) were assigned to this group. 28.6% of them suffered from diabetes and 71.4% had arterial hypertension. The subgroup ‘others’ comprises one patient with a syndrome of low intracranial CSF pressure due to shunt dysfunction.

### Abducens nerve palsies (CNP VI)

The abducens nerve palsy (CNP VI) represented the largest group with 210 patients (40%)—114 men and 96 women. 57 CNP VI (27.1%) were classified as idiopathic, 45.6% of them with a reported sudden onset.

In contrast to CNP IV, diabetes was only the fourth leading underlying cause of CNP VI, accounting for 13.3% of the patients. 78.6% with diabetogenic CNP VI described a sudden onset of symptoms.

Like for CNP IV, the most common underlying cause of CNP VI was inflammation with 30% of all cases (63 patients). Of these, 50 patients had an autoimmune/inflammatory disease and 13 patients had a defined viral or bacterial infection. The autoimmune/inflammatory diseases are represented by multiple sclerosis (*n* = 24), myasthenia gravis (*n* = 6), Tolosa–Hunt syndrome (*n* = 5), Miller-Fisher syndrome (*n* = 4), giant-cell arteritis (*n* = 2) and rarer diseases such as neurosarcoidosis, rheumatoid arthritis, ocular myositis, ANCA-positive vasculitis, NMDA-encephalitis. Viral infections were more frequently responsible for palsies than bacteria and were predominantly zoster radiculitis (*n* = 4). The remaining patients included infections such as neurosyphilis (*n* = 1), viral meningoencephalitis (*n* = 4), Lyme disease (*n* = 2), CNS-toxoplasmosis in an AIDS patient (*n* = 1) and a dacryocystitis with secondary spread into the orbita (*n* = 1).

Space-occupying lesions were also an important cause for CNP VI and responsible for 39 cases (18.6%). Two of them were located within the orbita, 28.2% were in or close to the cavernous sinus, including pituitary macroadenoma (*n* = 4), a carotid artery aneurysm (*n* = 1), sphenoidal sinusitis (*n* = 2) and a sinunasal carcinoma with infiltration of the cavernous sinus (*n* = 1). Furthermore, cavernous-sinus-fistulas (*n* = 3) were also detected as a cause. The remaining space-occupying lesions were the following: Intracranial hypertension (*n* = 5) was comparatively frequent, but also meningeosis carcinomatosa (*n* = 6), venous sinus thrombosis (*n* = 4), subarachnoid bleeding (*n* = 3), a pontine bleeding with perifocal edema (*n* = 1) and various neoplasms, such as metastases (*n* = 2), squamous cell carcinoma (*n* = 2), clivus chordoma (*n* = 1) and brainstem tumours (*n* = 2) were observed.

Only 7.1% of CNP VI were assigned to brainstem infarctions (*p* = 0.0004). All these patients described a sudden onset of the palsy and had an arterial hypertension, in addition, 13.3% had diabetes. The subgroup ‘others’ comprised seven patients with traumatic CNP VI (*n* = 4), iatrogenic CNP VI due to dental treatment (*n* = 1) and three patients with a syndrome of low intracranial CSF pressure.

### Multiple ocular motor nerve palsies

Multiple OMNPs were less common than isolated OMNPs, i.e., only 9.2% of our total cohort. Combined lesions of the third and sixth CN were most common (6% of the total cohort), while a combination of a CNP IV and CNP VI was rare (0.4% of the total cohort). If multiple OMNPs are observed, the chance to assign them to a defined etiology is much higher than for isolated OMNPs: only three patients had to be classified as idiopathic (6.5%).

More than every fifth combined palsy was due to a space-occupying lesion (23.9%). A clear majority of 72.7% had these lesions in or nearby the cavernous sinus or the orbita. The main space-occupying lesions were pituitary macroadenoma (*n* = 2), internal carotid artery aneurysms (*n* = 2), meningeosis carcinomatosa (*n* = 1), an isolated metastasis behind the orbita (*n* = 1) and different neoplasms (*n* = 5) such as meningioma, squamous cell carcinoma, sinunasal carcinoma, and an orbita tumour.

More than two thirds of the patients (67.4%) with combined OMNP had an inflammatory cause. 25 patients had an autoimmune/inflammatory etiology, whereas the remaining 6 OMNPs were of viral or bacterial origin. The autoimmune/inflammatory disorders comprised myasthenia gravis (*n* = 6), Tolosa–Hunt syndrome (*n* = 6), Miller-Fisher syndrome (*n* = 4), multiple sclerosis (*n* = 3), endocrine ophthalmopathy (*n* = 1), neurosarcoidosis (*n* = 1) and others. The viral infections were mainly diseases caused be the varicella-zoster-virus (*n* = 3). The remaining three patients had viral meningoencephalitis (*n* = 2) and an ascending bacterial infection of a purulent tooth (*n* = 1). Only one patient had a brainstem infarction as an underlying cause for a combined OMNP.

### Differences in etiologies between older and younger patients

**Fig. 3:**
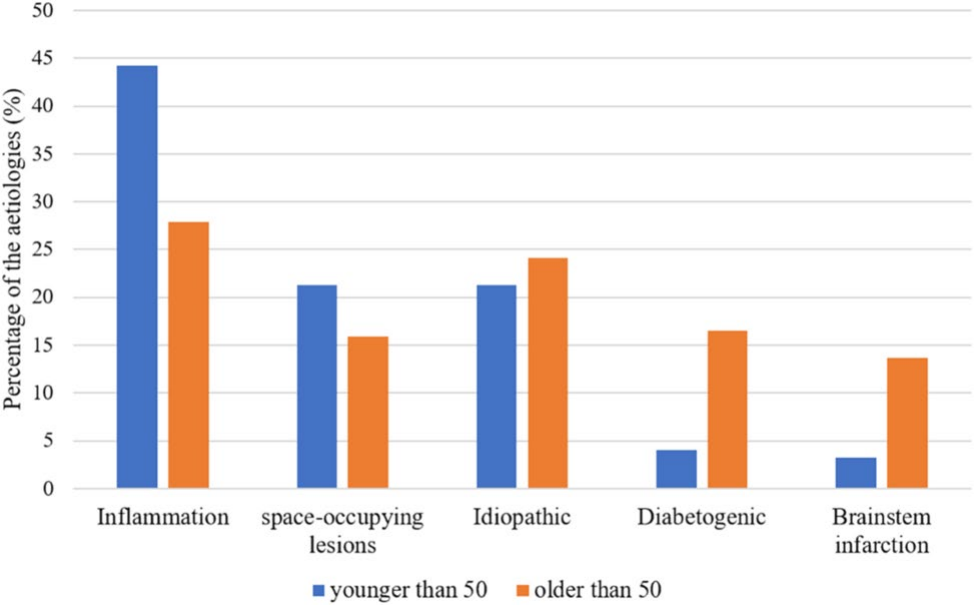
Proportion of etiologies in two different age groups: older than 50 years versus younger than 50 years

We also addressed the question whether the causes of OMNP vary in different age groups. In summary, vascular etiologies such as ischemic brainstem infarctions and diabetogenic OMNPs were significantly more common in patients over 50 years of age (diabetogenic *p* = 0.0005; brainstem infarction *p* = 0.0016). The incidences of space-occupying lesions and inflammations were higher in younger patients, but only the difference in inflammatory causes was significant (*p* = 0.0008). The age-related occurrence of idiopathic OMNPs decreased for CNP IV and CNP VI with age but increased in CNP III. Figure 3 summarizes the results.

### Involvement of other CNPs

127 of 474 patients (26.8%) had additional CNPs other than OMNPs, in particular facial nerve palsies (CNP VII), trigeminal nerve palsies (CNP V) or an affection of the opticus nerve (CN II). The proportion of inflammatory and space-occupying processes increased as soon as another nerve was additionally affected. One in three patients with an inflammation and 39% of the patients with space-occupying lesions exhibited an affection of an additional cranial nerve.

7.8% of this group (*n* = 37) had an additional affection of the trigeminal nerve; of these, 54% had an inflammatory etiology (*n* = 20) and 32.4% a space-occupying lesion (*n* = 12). Half of all inflammations were located intraorbitally or at the cavernous sinus, another 35% at the radix of the nerve. The dominant entities were multiple sclerosis, VZV-infections and THS. 58.3% of the space-occupying lesions were intraorbital or close to the cavernous sinus and only comprised aneurysms (*n* = 3) and tumours (*n* = 4).

8.7% of this group (*n* = 41) had CNP VII (30% had an inflammatory process (*n* = 16), 40% a space-occupying cause (*n* = 7) and 19.5% a brainstem infarction (*n* = 8)). The most common inflammations were multiple sclerosis (*n* = 4), neurosarcoidosis (*n* = 2) and radiculitis (*n* = 5)—the space-occupying entities were meningeosis carcinomatosa (*n* = 3), squamous cell carcinoma (*n* = 2), meningioma (*n* = 1) and thalamic bleeding with an edema (*n* = 1).

## Discussion

**Fig. 4:**
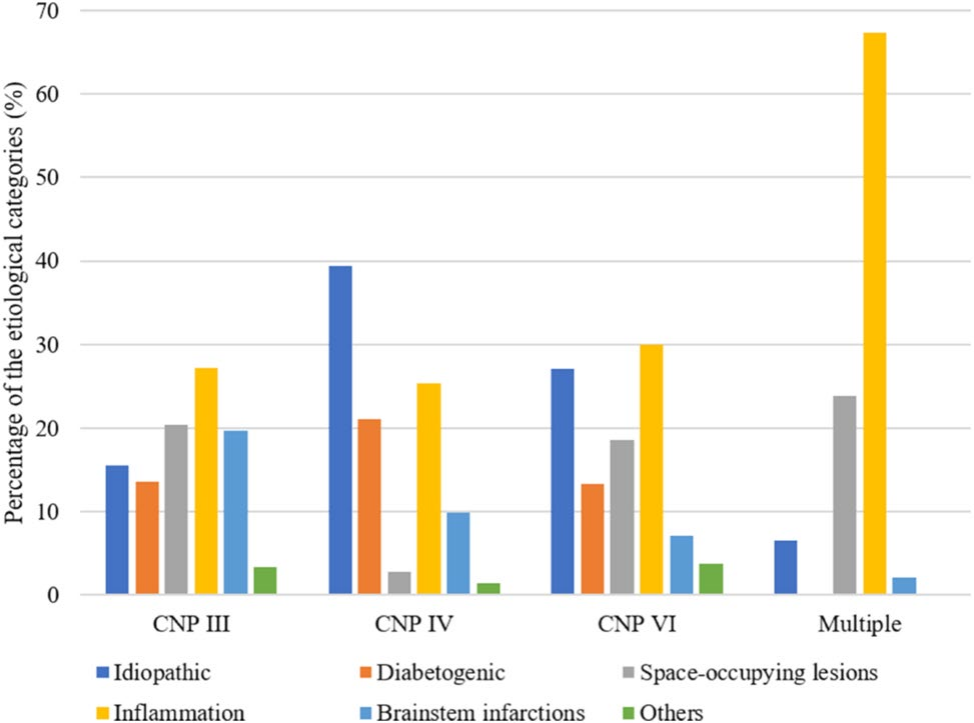
Proportion of the etiological categories in patients with CNP III, CNP IV, CNP VI, and multiple OMNPs

In this study of 504 patients admitted to the emergency unit of a Neurological University Clinic we analysed the assignment of oculomotor palsies to specific etiologies with the help of modern diagnostic tools, such a MRI and—if possible—CSF analysis. Abducens nerve palsies represented with 40% the most frequent single OMNP in our patient cohort which is largely consistent with the literature [1, 3, 6]. As in a previous study we less frequently observed multiple OMNPs (9.1%) [6]. A direct comparison with older studies was difficult, because often, different etiological categories were used. Inflammations for example, which played an important role in our study, were rarely mentioned as a separate etiological group in previous studies. In Fig. 4, the distribution of the etiological categories within the individual OMNPs is shown.

The most common cause of CNP III in our study were inflammations (27.2%), followed by space-occupying lesions (20.4%) and brainstem infarctions (19.7%). Only 15.7% of CNP III could not be attributed to any cause; therefore, significantly less palsies were described as idiopathic than in CNP IV (*p* = 0.0001) and CNP VI (*p* = 0.0104). The number of idiopathic palsies was lower than in most older studies [1, 3, 11, 12], but there were also studies that classified only 10–11% of the CNP III as idiopathic [6, 9], whereas we found significantly more inflammations and brainstem infarctions than in these studies [6, 9].

CNP IV had the highest rate of idiopathic (39.4%) and diabetogenic (21.1%) etiologies compared to the other nerves, but only the incidence of idiopathic CNP IV was significantly different from CNP III (*p* = 0.0001). Surprisingly, the percentage of idiopathic CNP IV in our study was higher than in older studies [3, 6]. Inflammatory causes were found in 25.4% and brainstem infarctions in 9.9% of CNP IV in our study. Therefore, we found more inflammations and brainstem infarctions in CNP IV than Choi et al. [6].

In CNP VI, inflammations were also the most common etiological category (30%) and were much more frequent if compared to the literature [6, 10]. Here, 27.1% of CNP VI had to be considered as idiopathic, another 18.6% were caused by space-occupying lesions and 7.1% by brainstem infarctions. Our results concerning idiopathic CNP VI were broadly consistent with older studies [10, 12], whereas we found more brainstem infarctions [6] similar to the other nerves studied.

It is interesting to note that in multiple OMNP, two thirds of the palsies were caused by inflammation (67.4%) and another 23.9% by space-occupying lesions. Only 6.5% of the palsies were classified as idiopathic and none as diabetogenic. A similarly high percentage of idiopathic and inflammatory multiple OMNPs were also found by Choi et al. [6].

2.1% of the total cohort (*n* = 10) had an aneurysm which led to a palsy due to nerve compression. We observed that four of these ten patients had mydriasis and they had an aneurysm close to the cavernous sinus. Not unexpectedly, the CN III was affected in eight out of ten cases (in two cases combined with another OMNP). The CN III is much more susceptible to aneurysms than the other OMNPs. For this reason, imaging of the skull is essential in the acute situation to exclude aneurysms. For aneurysm detection, the use of MRI and analysis of cerebrospinal fluid was sufficient in our study. In case of doubt, we rarely used also a CT-scan.

It was a major difference to the literature that we were able to identify a high number of brainstem infarctions, especially for CNP III (19.7%). Brainstem infarctions were significantly less common in older studies [6, 9, 10, 13], for instance, Chou and colleagues found brainstem infarctions in only 3.5% of CNP III [13]. This discrepancy in the distribution of the underlying etiologies could be due to technological advances in neuroimaging, i.e., standard use of MRI, which enabled us to make precise diagnoses and led to an increase in the prevalence of ischaemic causes. The OMNPs due to brainstem infarctions were always accompanied by additional neurological symptoms (such as ataxia or dysphagia). Interestingly, the frequency of brainstem infarctions varied greatly between the individual nerves in our study. CNP III was caused more often by brainstem infarctions than CNP IV (*p* = 0.659) and VI (*p* = 0.0004). One possible explanation would be that the area of the nucleus of the third cranial nerves nucleus is the largest one and a comparatively long part of its axon is located in the brainstem.

Despite of up-to-date diagnostic procedures, the percentages of idiopathic palsies in our study were somewhat higher than in the majority of the literature. An explanation for this puzzling result lies in the fact that we defined stricter etiological categories than in these older studies. For example, we did not use a definition of vascular etiology that was previously and commonly used. This definition consisted of indirect indicators of vascular disease, such as the risk factors age, hypertension or diabetes. In this study we tried to define the vascular etiology more precisely. For this purpose, we used the category of brainstem infarctions, which is only applied if there was definite MRI evidence of ischaemia in the pons or mesencephalon. We also defined the category of diabetogenic OMNP which was based on laboratory evidence for the presence of diabetes mellitus.

Patients with diabetes who could not be attributed to any other cause were assigned to diabetogenic OMNPs. Any patient without diabetes for whom no cause could be found was considered as idiopathic, regardless of existing risk factors such as hypertension or old age. This strict classification explains why the idiopathic category is more frequently represented in our study. The designation of a diabetogenic OMNP remains strictly speaking not more than an additional exclusion diagnosis, since its underlying pathomechanism is still uncertain. There is some debate whether the cause is due to vascular diseases [14–16].

In our study, far more patients had inflammations than described in the literature. Depending on the nerve, inflammation accounted for 25–30% of etiologic factors. In the literature, the percentages are often well below 20% [6, 9, 10]. This might be explained by the routine use of a CSF analysis in the majority of patients, i.e., more frequently than usually reported in older studies. The percentage of inflammation did not differ between the three nerves. The frequency of the individual inflammatory entities, on the other hand, varied greatly in some cases. For example, we found multiple sclerosis (MS) predominantly in patients with CNP VI representing 70.6% of the MS cases encountered. It is known in the literature that CNP VI is very common in MS [17]. The Tolosa–Hunt syndrome (THS) was also rather common in our population (5.5%). In the literature, granulomatous inflammatory processes between the orbita and the cavernous sinus are often described as causes [18]. 57.7% of all THS cases in our study showed inflammation on MRI, in 86.7% of these cases the inflammation was located retroorbitally or at the top of the orbita. In our cohort ocular pain was reported in 67 patients (14.14%), of whom 24 patients (35.82%) had Tolosa–Hunt syndrome. It was another interesting result that half of all THS patients showed an isolated CNP III. Since the oculomotor nerve branches retroorbitally several times, this could be an explanation for the increased vulnerability of the nerve.

In the following, we focused on the patients with multiple cranial nerve palsies (26.8% of the cohort); here, the etiologies of inflammation and space-occupying lesions were overrepresented if compared to single nerves. In addition, the proportion of idiopathic palsies in patients with multiple CNP was only 9.8% (total cohort: 23.4%). A reason why multiple CNP can be assigned to a defined etiology more frequently than isolated OMNP might be, that the involvement of multiple nerves indicates the presence of larger lesions. However, it was also obvious, that an additionally affected nerve can provide more information about its location of the cause in the brain. For example, CNP III is closest to CN II and CN V at the cavernous sinus and intraorbitally, so a lesion would be anatomically most plausible there. In fact, two thirds of CNP III with an additional CNP II or V were located there. Another 15% were caused by an anatomically defined lesion in the brainstem or at the root. Every eighth patient (12.2%, *N* = 18) also had a palsy of the seventh nerve (CNP VII), who enters the inner auditory canal directly after exiting the brainstem. Therefore, the anatomical proximity between CNP III and VII can only be considered inside the brainstem or directly after its exit. Supporting the hypothesis, 61.1% of the lesions could be ascribed to the brainstem or the roots and neighbouring meninges, when both nerves where affected. Another 16.7% had an underlying systemic disease such as multiple sclerosis.

One fifth of our patients with CNP VI had additional lesions of CN II (6.2%), CN V (9.5%) or CN VII (7.6%). This is easily explained by the anatomical proximity of the second and fifth cranial nerve close to the cavernous sinus and intraorbitally. 34.4% of the causes were located here. However, the percentage of causes was significantly higher at 31.3% in the brainstem and the roots and nearby meninges. The other causes were mainly systemic diseases (18.8%) such as MS, pseudotumor cerebri and myasthenia gravis. The well-known anatomical proximity of the sixth and seventh cranial nerves is the *inner facial knee*, which runs around the abducens nucleus. A quarter of the causes were located there. Due to the early intraosseous disappearance of CN VII, only a short anatomical proximity remains after leaving the brainstem. Therefore, 43.8% of the patients with CNP VI and VII suffered from meningitis, radiculitis, meningeosis carcinomatosa or neoplasm at the clivus.

A limitation of our study is the obvious referral bias, due to a patient population which is specific to an emergency room which is devoted to neurological patients. For example, the etiologies of OMNP in an ophthalmologic clinic may differ from neurological departments. Furthermore, in our study, due to the retrospectively collected data, a follow-up analysis was only possible in an insufficient number of patients, which is why we excluded these data from the study. Therefore, in individual cases it may be possible that patients who were initially classified as idiopathic or diabetogenic, might be reclassified a few months later. Data regarding individual symptoms (such as pathological corneal reflex) were often not complete and are not presented in this paper.

## Conclusion

The causes of OMNPs are very heterogeneous, ranging from neoplasms, aneurysms and brainstem infarctions to autoimmune diseases, viral/bacterial infections or trauma. However, even if diagnostic tools such as MRI and CSF analysis are routinely applied according to a prespecified protocol, up to a quarter of the cases remained unexplained. However, the use of these modern diagnostic tools enabled us to diagnose the causes more precisely. As a result, the inflammatory and ischaemic causes of OMNP were observed to be much more frequent than previously reported. We found that about one fifth of CNP III were caused by brainstem infarctions, for which a MRI was essential. We were also able to show that evidence for an additional CNP, especially those of the second, fifth or seventh CN, can serve as a valuable information on the location of the lesion. Furthermore, if an additional nerve is affected the etiological profile changed strongly in the direction to inflammation and space-occupying lesions. Because multiple nerve involvement often means the presence of a larger lesion, these are more likely to be detected on MRI. The use of modern diagnostic tools is the basis for future interventional trials.

## References

[1] Rucker C (1958). Paralysis of the third, fourth, and sixth cranial nerves. J Ophthalmol.

[2] Khawam E, Scott AB, Jampolsky A (1967). Acquired superior oblique palsy: diagnosis and management. JAMA Ophthalmol.

[3] Richards BW, Jones FR, Younge BR (1992). Causes and prognosis in 4278 cases of paralysis of the oculomotor, trochlear, and abducens cranial nerves. Am J Ophthalmol.

[4] Kung NH, Stavern GP (2015). Isolated ocular motor nerve palsies. Semin Neurol.

[5] Danieli L, Montali M, Remonda L (2018). Clinically directed neuroimaging of ophthalmoplegia. Clin Neuroradiol.

[6] Choi KD, Choi SY, Yang TH (2019). Aquired ocular motor nerve palsy in neurology clinics: a prospective multicenter study. J Clin Neurol.

[7] Elder C, Hainline C, Galetta SL (2016). Isolated abducens nerve palsy: update on evaluation and diagnosis. Curr Neurol Neurosci Rep.

[8] Murchison AP, Gilbert ME, Savino PJ (2011). Neuroimaging and acute ocular motor mononeuropathies. Arch Ophthalmol.

[9] Berlit P, Reinhardt-Eckstein J, Krause KH (1988). Zur Ätiologie, Prognose und Therapie der isolierten Oculomotoriusparesen. Nervenarzt.

[10] Berlit P, Reinhardt-Eckstein J, Krause KH (1989). Die isolierte Abduzensparese - eine retrospektive Studie an 165 Patienten. Fortschr Neurol Psychatr.

[11] Green W, Hackett E, Schlezinger N (1964). Neuro-ophthalmologic evaluation of oculomotor nerve paralysis. Arch Ophthalmol.

[12] Kim K, Noh SR, Kang MS (2018). Clinical course and prognostic factors of acquired third, fourth and sixth cranial nerve palsy in Korean patients. Korean J Ophthalmol.

[13] Chou KL, Galetta SL, Liu GT (2004). Acute ocular motor mononeuropathies: prospective study of the roles of neuroimaging and clinical assessment. J Neurol Sci.

[14] Dreyfus P, Hakim S, Adams R (1957). Diabetic ophtalmoplegia: report of case, with postmortem study and comments on vascular supply of human oculomotor nerve. AMA Arch Neurol Psychiatry.

[15] Hopf H, Gutmann L (1990). Diabetic 3rd nerve palsy: evidence for a mesencephalic lesion. Neurology.

[16] Thömke F, Tettenborn B, Hopf H (1995). Third nerve palsy as the sole manifestation of midbrain ischemia. J Neuroophthalmol.

[17] Thömke F, Lensch E, Ringel K (1997). Isolated cranial nerve palsies in multiple sclerosis. J Neurol Neurosurg Psychiatry.

[18] Sahlmüller M, Schroeter J (2011). Idiopathische entzündliche Orbitopathie. Klin Monbl Augenheilkd.

